# Triple‐reassortant influenza A virus with H3 of human seasonal origin, NA of swine origin, and internal A(H1N1) pandemic 2009 genes is established in Danish pigs

**DOI:** 10.1111/irv.12451

**Published:** 2017-03-21

**Authors:** Jesper Schak Krog, Charlotte Kristiane Hjulsager, Michael Albin Larsen, Lars Erik Larsen

**Affiliations:** ^1^ National Veterinary InstituteTechnical University of DenmarkFrederiksberg CDenmark; ^2^ Merial Norden A/SHørsholmDenmark

**Keywords:** case report, H1N1pdm09, H3N2, influenza A virus, zoonosis

## Abstract

This report describes a triple‐reassortant influenza A virus with a HA that resembles H3 of human seasonal influenza from 2004 to 2005, N2 from influenza A virus already established in swine, and the internal gene cassette from A(H1N1)pdm09 has spread in Danish pig herds. The virus has been detected in several Danish pig herds during the last 2‐3 years and may possess a challenge for human as well as animal health.

## 1 Introduction

Influenza A viruses (IAV) are important human and animal pathogens with high impact on public and animal health. The virus contains eight separate RNA segments of which the hemagglutinin (HA) and neuraminidase (NA) segments define the subtype. In case of mixed infection with different viruses, exchange of segments may lead to a new reassortant virus. Such reassortant viruses occasionally emerge with a mixture of genetic material from different species. Pigs are considered a mixing vessel for the generation of such viruses with genetic material originating from pigs, humans, and/or birds.^1^

Until 2009, H1N1, H1N2, and H3N2 were the dominating enzootic subtypes of influenza A virus in swine (swIAV) in Europe. These subtypes all have genetic and antigenic characteristics that differ from the human seasonal influenza virus subtypes H1N1 and H3N2.^2^ In 2009, a new human pandemic strain (A(H1N1)pdm09) entered the global swine population by a reverse zoonotic event. This virus is now circulating in both the human and swine populations and reassortant viruses, with mixtures of A(H1N1)pdm09 and enzootic swIAV origin genes, which have been frequently recovered.^1^, ^3^, ^4^ Reassortant viruses with genes from other human seasonal IAV origin and enzootic swIAV are occasionally detected in pigs. Remarkably, these reassortments are typically detected in pigs several years after their human counterpart appeared as seasonal influenza viruses.^5^, ^6^, ^7^

In Denmark, a passive surveillance program for swIAV has been conducted since 2011 based on samples submitted for diagnostic purpose from pigs with acute respiratory diseases. The most frequent circulating subtypes are avian‐like H1N1 (avH1N1), reassortant IAV with avian‐like H1 and internal gene cassette, and with N2 from swine‐adapted H3N2 (rH1N2) and A(H1N1)pdm09.^8^ In addition, a number of different reassorted viruses are found sporadically.^1^ As a part of the Danish surveillance of influenza viruses in swine, a new reassortant influenza A virus with an HA gene which clusters in the clade of seasonal human H3N2 viruses from 2004 to 2005 and the NA gene closely related to Danish rH1N2 viruses was detected in a single herd.

In this report, the result of full genome characterization of this new swine virus subtype is presented. The virus is distinct from other contemporary European swine H3N2 viruses and from the strains included in commercially available vaccines for use in pigs. In 2015/2016, the virus was detected in several other herds indicating that this new subtype has been established in the Danish pig population.

## 2 Case Description

In 2014, samples from a herd located in the central part of Jutland, Denmark, were submitted to the National Veterinary Institute, due to persistent problems with respiratory disease in pigs and reproductive problems in the sow herd. The herd vaccinated sows with the Gripovac3 vaccine (Merial Norden). This vaccine contains three enzootic swine influenza strains: an avian‐like H1N1 (A/sw/Haselunne/IDT2617/2001(H1N1)), a H1N2 strain with a human‐like H1 (A/sw/Bakum/1832/2000 (H1N2)), and a H3N2 strain (A/sw/Bakum/IDT1769/2003)). IAV was detected in nasal swabs from piglets by an in‐house‐modified real‐time RT‐PCR (rtRT‐PCR) targeting the matrix segment.^9^ Initial capillary electrophoresis sequencing of the HA and NA segment was performed on the amplicons made using modified primers designed by Hoffmann et al.^10^ Analysis of the sequences by BLAST search of the EpiFlu database and phylogenetic analysis revealed that closest hit in a BLAST search (95% identity) was to the H3 genes from the human seasonal influenza virus circulating in 2004/05 (designated H3hu05). NA was phylogenetically closely related (~97% identity) to that of rH1N2 viruses routinely found in Danish pigs (designated N2sw). The virus was named: A/swine/Denmark/1916‐1/2014 (H3hu05N2sw). To our knowledge, this is the first case in Europe of spillover of human H3 surface genes that have been established and found to circulate in swine.

**Figure 1:**
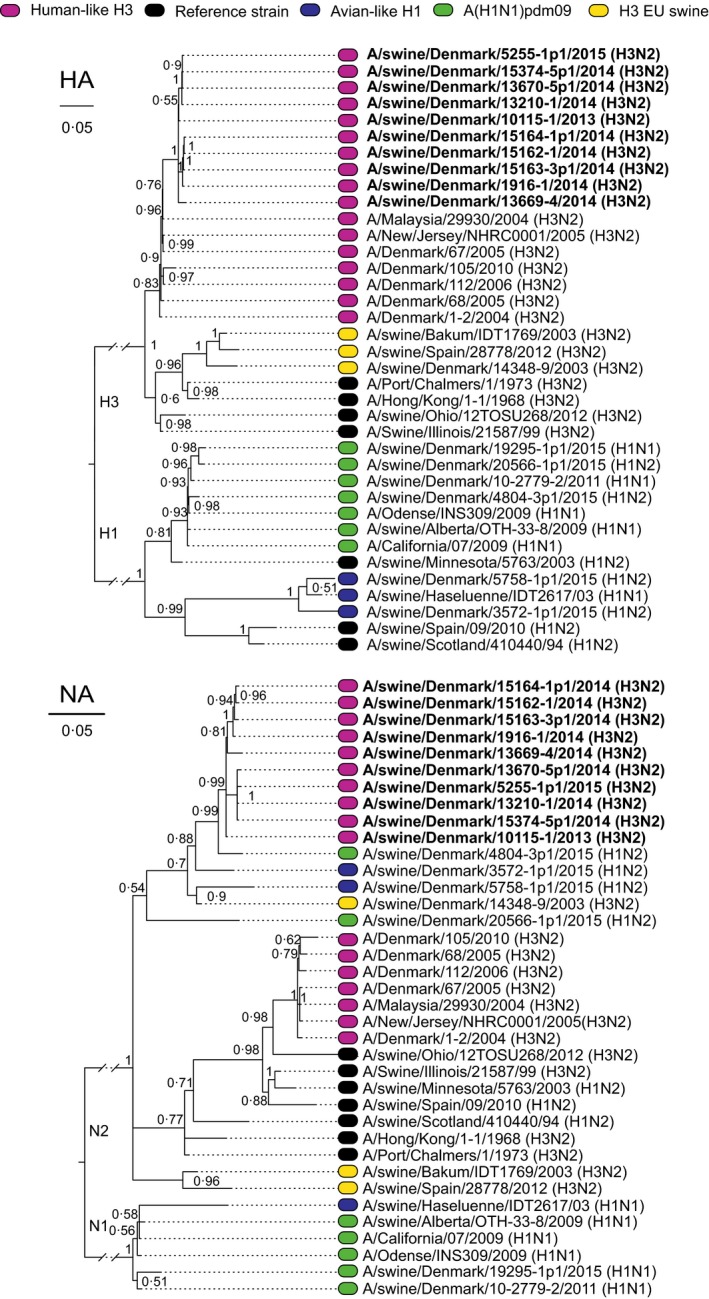
Phylogenetic tree of HA (top) and NA (bottom) nucleotide sequences from the survey along with reference strains. The scale denotes substitutions per site. For sample information, please refer to Table 1

**Figure 2:**
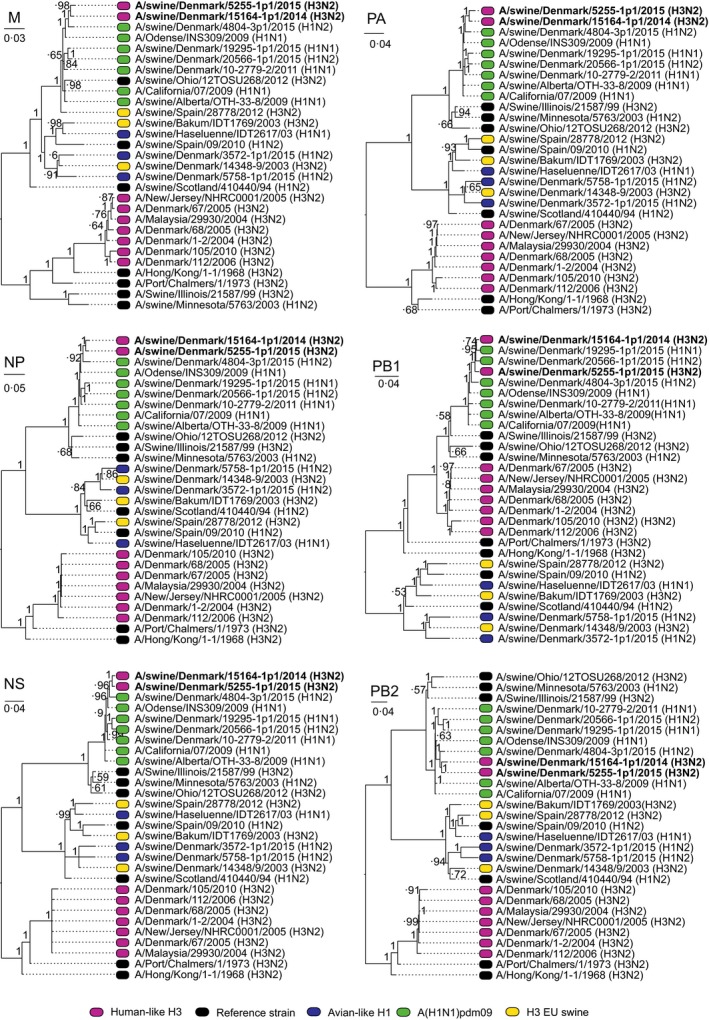
Phylogenetic tree of the six internal genes of the two isolates that were fully characterized together with reference strains. The coloring is dictated by the HA tree in Figure 1

To investigate whether this new subtype circulated prior to 2014, samples from 2011 to 2014 were retrospectively assessed and these showed that a virus (A/swine/Denmark/10115/2013) with the H3hu05 HA was already present in samples from 2013. Like A/swine/Denmark/1916‐1/2014 (H3hu05N2sw), it was not possible to propagate this virus on MDCK cells or embryonated chicken eggs, and therefore, it was unclear whether this was a reassortant or just a mixture of different viruses in the same sample. However, this 2013 virus originated from a herd that was situated in the same geographical area as the index case. The two herds had two different owners (A and B). Each owner had three distinct production sites. To investigate whether the reassortant virus had spread among these six sites, five pools consisting of 10 nasal swabs from 10 different pigs were sampled. Preferable pigs with respiratory signs were sampled, but if no clinical signs were observed, the youngest pigs present were sampled (samples included pigs aging 2‐6 weeks). Pools were collected from all six production sites and tested for IAV by rtRT‐PCR as above. From positive samples, the HA/NA genes were sequenced by capillary electrophoresis as described above. To further characterize the virus and confirm the subtype, isolation in MDCK cells was performed. Full genome sequencing was performed on two cell culture isolates (A/swine/Denmark/15164‐1p1/2014 (H3hu05N2sw) and A/swine/Denmark/5255‐1p1/2015) one from each owner. All eight gene segments were amplified utilizing in‐house‐designed primers using SuperScript III One‐Step RT‐PCR System with Platinum Taq High Fidelity. Purified PCR products were pooled in equimolar quantity and subjected to next‐generation sequencing on the Ion Torrent PGM™ sequencer. The sequences were assembled using CLC genomics workbench 6.5 (Qiagen), and phylogenetic trees were constructed with MrBayes 3.2.6^11^ using the CIPRES science gateway^12^ from alignments constructed with the MUSCLE algorithm. For all segments, the following parameters were used nset=6, rates=invgamma, ngen=10 000 000 or to convergence of 0.002. The analysis showed that samples from both owners were closely related with 97%‐100% nt identity (Figure 1) but clustering based on owner is also observed, with more than 99% identity when comparing sequences obtained from one owner with exception of sample A/swine/Denmark/13669‐4/2014 that had ~97% identity to all other sequences. The analysis also revealed that all the internal genes shared >98% identity to genes of circulating A(H1N1)pdm09 porcine strains (Figure 2). In Table 1, a complete summary of results of each herd are shown including the EpiFlu accession numbers.

**Table 1:** Overview of the samples used in this project

Owner‐site	Name	Samples (pools) (Ct)					DS	Isolate	NGS	EpiFlu
		1	2	3	4	5				
A‐1	A/swine/Denmark/15164‐1p1/2014	**19**	36	29	25	22		x	x	EPI906241‐8
A‐2	A/swine/Denmark/15163‐3p1/2014	24	34	**20**	24	21		x		EPI906257‐8
A‐3	A/swine/Denmark/15162‐1/2014	28	neg	neg	neg	36				EPI906569‐70
A‐3	A/swine/Denmark/1916‐1/2014						x			EPI906259‐60
B‐1	A/swine/Denmark/13669‐4/2014	28	neg	31	23	31				EPI906571‐2
B‐1	A/swine/Denmark/10115‐1/2013						x			EPI906261‐2
B‐2	A/swine/Denmark/13670‐5p1/2014	23	22	36	neg	**20**		x		EPI906573‐4
B‐2	A/swine/Denmark/13210‐1/2014						x			EPI906575‐6
B‐3	A/swine/Denmark/15374‐5p1/2014	neg	29	34	29	**24**		x		EPI906577‐8
B‐3	A/swine/Denmark/5255‐1p1/2015						x	x	x	EPI906249‐56

DS, Diagnostic sample, sent in for routine diagnostic and not collected for this study; NGS, Samples (x) that were fully sequenced. NGS denotes the samples where the internal gene cassette was also sequenced. Ct in bold represents the sample used for virus isolation.

According to information in the Danish database on movement of pigs (www.svineflyt.dk), the two production systems did not exchange pigs at any time from June 2005 to 2015, did not have contact with each other, and had different staff and veterinary practitioners.

In 2015, another sample from owner B was submitted for test, and this sample was again positive for H3hu05N2sw (A/swine/Denmark/5255‐1p1/2015). The herd had experienced severe clinical signs including reproductive failures despite the fact that the sows were vaccinated with Gripovac 3 (Merial Norden). As the vaccine seemed to fail to protect the animals against severe clinical signs, hemagglutination inhibition (HI) tests were performed to assess cross‐reactivity. Viruses from the two different production systems: A/swine/Denmark/5255‐1p1/2015 (H3hu05N2sw) and A/swine/Denmark/15164‐1p1/2014 (H3hu05N2sw), were tested along with A/swine/Denmark/10115/2007 (H3N2) which is an older Danish enzootic swine‐adapted H3N2 strain. The sera tested included serum from two pigs vaccinated with Gripovac 3 (Vacc1, Vacc2), serum from five sows from the herd where A/swine/Denmark/15164‐1p1/2014 was isolated (H3hu1‐H3hu5), and a swine H3N2 control serum used in the routine diagnostic laboratory at the National Veterinary Institute (H3ref; Table 2). The results revealed that there was no cross‐reactivity between post‐vaccination serum and the H3hu05N2sw isolate (Table 2). This was expected based on the relatively large difference (81%‐82% amino acid identity) in the HA sequence between the H3hu05N3sw viruses and the H3 strain included in the vaccine.^2^

**Table 2:** Hemagglutination results showing titers between the antigens and sera used

Antigen	Sera							
	H3hu1	H3hu2	H3hu3	H3hu4	H3hu5	Vacc1	Vacc2	H3ref
A/swine/Denmark/15164‐1p1/2014 H3huN2sw	80	80	160	160	80	—	—	—
A/swine/Denmark/5255‐1p1/2015 H3huN2sw	40	80	160	160	40	—	—	—
A/swine/Denmark/10115/2007 H3N2	—	—	—	—	—	160	40	40

H3hu# denotes sera from five different sows from the herd infected with A/swine/Denmark/15164‐1p1/2014 H3huN2sw. Vacc# is post‐vaccination with Gripovac 3 serum provided by the manufacturer of the vaccine IDT. H3ref is the standard sera used by the National Veterinary Institute of Denmark routine diagnostic laboratory.

Test of samples received in the frame of the national surveillance program in 2015‐2016 has revealed that at least seven other production systems located geographically widespread in Denmark were positive for this novel subtype, indicating that this virus has been established in Danish swine (data not shown).

## 3 Conclusions

We report here the detection of a new triple‐reassortant H3N2 influenza virus in swine with H3 gene of seasonal human influenza virus origin, internal genes from A(H1N1)pdm09‐like viruses, and NA from contemporary N2 swine viruses. Its genetic makeup is distinct from previously known European swine H3N2 viruses, and the virus was retrospectively detected in a sample from 2013. It is now apparent that the subtype has become established in the Danish pig population. The reservoir of this virus during the period from 2004 to 2005 human influenza season until 2013 can only be speculated, as it has been reported neither in the human nor in any animal populations during that period. Therefore, further molecular clock analysis is needed on more isolates to elucidate when this virus emerged, and to confirm that the parent virus is indeed from the 2004 to 2005 human influenza season. The reassortment events leading to this virus also remain speculative. If the H3 gene indeed originated from the human seasonal influenza strains circulating in 2004/2005, this H3 gene have circulated undetected in swine or another host for more than 10 years, and until the emergence of A(H1N1)pdm09, this must have been without the internal genes derived thereof. In our view, a likely scenario is that the H3 gene reassorted with a swine influenza strain with an HXN2‐avian‐like backbone creating a virus that did not cause severe clinical signs. In 2010 or later, this virus reassorted with A(H1N1)pdm09 creating the H3hu05N2 virus which apparently is capable of inducing severe clinical signs in pigs. Denmark is annually exporting more than 10 million living pigs. As pigs are not routinely tested for IAV in relation to export, it is likely that this virus will spread to other European countries, emphasizing the need of joint European surveillance initiatives such as the former European Union funded ESNIP programs.^1^, ^8^

As there was no link between the two independent production systems, the introductions either happened independently from a third source or by transmission between the production systems by other horizontal routes, for example, airborne transmission.

The human‐like swine H3N2 virus is distinct from the strains included in all available swine vaccines in Europe and, furthermore, the prevalence of viruses in swine with an H3 gene is very low in pigs in most European countries including Denmark.^8^ Thus, the establishment of this new H3N2 virus in pigs could have a significant impact on the swine industry due to lack of population immunity. Indeed, the respiratory disease in pigs and reproductive failures in sows reported from some of the herds in this study were quite severe despite vaccination of the sows. According to the practitioners we have had contact with, the clinical sigs seen in the herds are comparable to or even more severe than the clinical signs normally encountered during acute outbreak of influenza in Danish swine, so this virus seems to be as virulent or even more virulent than the enzootic circulating strains. Further controlled studies are needed to address this further.

The identification of this new virus with seven of eight genes of human origin including an A(H1N1)pdm09 matrix gene also raises severe concern on the impact on human health. In the United States, swine‐adapted H3N2 viruses which also have acquired the A(H1N1)pdm09 matrix gene^13^ have been shown to be able to infect humans, albeit with limited human‐to‐human transmission. The new virus reported here has in addition to the A(H1N1)pdm09 matrix gene also a human‐adapted HA gene which may lead to an improved risk of human‐to‐human transmission if introduced into humans. Studies are ongoing to investigate these further using ferrets as infection models.
